# Dr Gururaj Nadig – A Tribute

**Published:** 2009

**Authors:** Prasanna Latha Nadig, Velayutham Gopikrishna

**29^th^ March 1957 – 25^th^ October 2009**


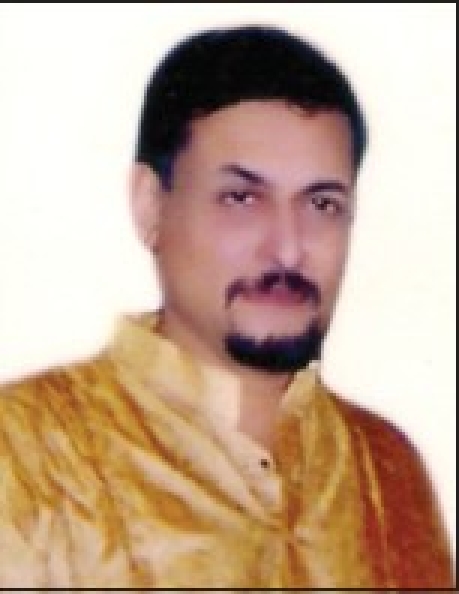



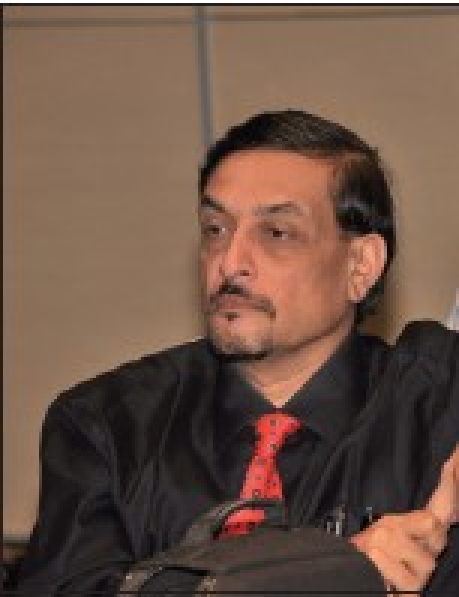


Dr. Gururaj Nadig was born on March 29, 1957, in Koppa and hails from the Shimoga district, Karnataka, India. He completed his schooling from Masur and his college days were spent in Hubli, Karnataka. He then joined Government Dental College, Bangalore for his B.D.S course and graduated in the year 1984 with a good academic record. He was the General Secretary of the college students' association. He completed his Masters in Conservative and Endodontics from the same college in the year 1989. Some of the significant papers presented by him included on Endodontic Implants and a case report on bicuspidization. He underwent an advance training course in Crown and Bridge Prostheses at the dental training centre in Wehrheim, Germany in the year 1992.

Dr. Gururaj Nadig had 22 years of teaching experience. He started his career as a tutor in St. John's Medical College in the dental department. He then worked as a lecturer, Assistant Professor, Professor and then the Head of the Department in V.S. Dental College. He later joined Rajiv Gandhi Dental College and worked as a Professor, Head of the Department of Conservative Dentistry and Principal (in-charge). He was working as a Professor and Head of the Department at Oxford Dental College, Bangalore, when he met his untimely demise. He had actively guided many post graduate students and was an astute academician.

Dr. Gururaj Nadig was an active member of Indian Dental Association from 1985 onwards and had worked in various capacities as a Joint Secretary of the local branch, Vice President and then as the President of the Bangalore branch during 1999–2000. During this period, he had conducted various dental camps at old age homes and handicapped homes.

He started his private practice in the year 1985 and worked as a consultant for Kudremukh Iron Ore Company and also as a chief consultant at Sri Bhagwan Mahaveer Jain Hospital. He was a recipient of *Karnataka Chaitanya* state level award for Excellence in Dentistry in the year 1999. He was the Chief Coordinator for dental programs conducted by Colgate-Palmolive India held in the urban and rural districts of Bangalore. He was a busy private practitioner and some of his elite patients included Shri H. D. Deve Gowda, former Prime Minister of India, Shri H. K. Patil, irrigation minister of Karnataka, sports personalities like Anil Kumble, cine actors like Vishnuvardhan and Ambarish to name a few.

He was an executive member of our society – FODI, and recently represented India at the Asian Endodontic Forum 2009 at Qingdao, China. Dr. Gururaj was a very dynamic person who never liked a dull day in his life. He was very systematic in whatever he did, liked people around him and enjoyed organizing programs and cultural events. He was a man of few words and believed in his own principles. He loved to live life stylishly and king size. He took immense pleasure in serving mankind. He had a charismatic personality and was popular amongst his friends who would always count on him at any time.

His sudden demise on October 25, 2009 due to a massive heart attack was a rude shock and has left a huge void in our fraternity. He leaves behind his wife Dr. Prasanna Latha, daughter Pooja and son Mayur. These are a few memories of a life remembered.

At birth, our lives are like a plain canvas…..
Our potential is the colors……
Our choices are the strokes on the canvas…….
At death, this canvas will either be a treasured masterpiece or an unnoticed scribbling…..
Dr Gururaj Nadig touched the canvas of many people and left behind indelible marks in the field of Conservative Dentistry & Endodontics….
Prasanna Latha Nadig, Velayutham Gopikrishna

